# A72 INFLUENCE OF THE MICROBIOME ON DENDRITIC CELL FUNCTION

**DOI:** 10.1093/jcag/gwae059.072

**Published:** 2025-02-10

**Authors:** W L Miller, A Kazanova, M Brouillard-Galipeau, C Gavino, S Gruenheid

**Affiliations:** Microbiology & Immunology, McGill University Faculty of Medicine and Health Sciences, Montreal, QC, Canada; Microbiology & Immunology, McGill University Faculty of Medicine and Health Sciences, Montreal, QC, Canada; Microbiology & Immunology, McGill University Faculty of Medicine and Health Sciences, Montreal, QC, Canada; Microbiology & Immunology, McGill University Faculty of Medicine and Health Sciences, Montreal, QC, Canada; Microbiology & Immunology, McGill University Faculty of Medicine and Health Sciences, Montreal, QC, Canada

## Abstract

**Background:**

The intestinal microbiome educates the immune system, in part through signaling in antigen- presenting cells including dendritic cells (DCs). Germ-free mice exhibit DC deficiency, migration impairment, and priming dysfunction, implying a relationship between microbiota and DC function. Naturally acquired, endemic Helicobacter species (H. spp.) such as H. hepaticus are common in many mouse colonies, whereas some facilities are Helicobacter-free.

**Aims:**

Although they do not cause overt disease in most immune-competent mouse strains, we hypothesized that H. spp. could be a source of low-level inflammation, resulting in immune reprogramming and leading to phenotypic differences between H. spp.-positive and H. spp.- negative mice.

**Methods:**

We generated bone marrow-derived dendritic cells (BMDCs) from H. spp.-positive mice and from genetically identical mice rendered H. spp.-free through mouse rederivation. BMDCs were stimulated for 6 hours (or not) with ultra-pure lipopolysaccharide (LPS). We measured expression of molecules MHC-I and MHC II (Signal 1 of antigen presentation) and CD80, CD86 and PD-L1 (Signal 2), the “activating” epigenetic mark of trimethylation at lysine 3 of histone 4 (H3K4me3) and metabolic changes via flow cytometry. Multiplex ELISA was used to measure secreted cytokines (Signal 3) and in vitro methods were used to assay BMDC antigen presentation. Bulk RNA sequencing of BMDCs was performed to further understand microbiome-linked changes in BMDC phenotype.

**Results:**

Surface expression of molecules related to Signal 1 and Signal 2 of antigen presentation were increased both at steady state and after LPS stimulation in BMDCs derived from H. spp.-positive in comparison with H. spp.-free mice and this correlated with the abundance of H3K4me3. The presence or absence of H. spp. also altered metabolic phenotype of BMDCs upon LPS stimulation.

**Conclusions:**

The presence of Helicobacter in the gut microbiome influences BMDC phenotype and may alter BMDC function. We anticipate epigenetic changes involved in BMDC phenotypic and functional changes, which will require in-depth epigenetic profiling. Future directions also include fecal microbiota transplant experiments and Helicobacter infection models followed by BMDC characterization using our established pipeline.

**Funding Agencies:**

**None**

